# Evidence summary on the rehabilitative management of dysphagia during radiotherapy for head and neck cancer patients

**DOI:** 10.3389/fonc.2024.1429484

**Published:** 2024-09-09

**Authors:** Yu Zhang, Hongwei Wan, Yu Zhu, Shuman Wang, Mimi Zheng, Xiaoru Li

**Affiliations:** ^1^ Department of Nursing, Shanghai Proton and Heavy Ion Center, Fudan University Cancer Hospital, Shanghai, China; ^2^ Shanghai Key Laboratory of Radiation Oncology, Shanghai, China; ^3^ Shanghai Engineering Research Center of Proton and Heavy Ion Radiation Therapy, Shanghai, China

**Keywords:** head and neck neoplasms, radiotherapy, dysphagia, rehabilitation, evidence summary

## Abstract

**Objective:**

To retrieve, extract, integrate and evaluate evidence on the rehabilitation of dysphagia in patients undergoing radiotherapy for head and neck cancer (HNC), and to provide a basis for the development of a rehabilitation management protocol for dysphagia in patients undergoing radiotherapy for HNC.

**Methods:**

An evidence-based systematic search of the literature related to the rehabilitation of dysphagia in patients with HNC during radiotherapy was conducted from January 2013 to March 2023, and the corresponding evaluation tools were selected according to the different types of literature for quality evaluation. “The Joanna Briggs Institute (JBI) evidence pre-grading system was used to evaluate the quality of the evidence.

**Results:**

A total of 17 articles were included, including 3 guidelines, 5 expert articles, 1 clinical decision, 1 practice recommendation, 2 evidence summaries and 5 systematic evaluations. A final total of 28 pieces of evidence were summarised, including 6 areas of swallowing disorder screening and assessment, physiotherapy, preventive swallowing function training, feeding management, pain control, and oral care.

**Conclusion:**

This study forms a multidisciplinary collaborative evidence summary for the rehabilitation management of dysphagia in patients undergoing radiotherapy for HNC, but the application of some of the evidence needs to be carried out in the context of the clinical setting and patient-specific circumstances for the rehabilitation evidence selected for patients’ dysphagia to improve their swallowing function and their swallowing-related quality of life and reduce the occurrence of related complications.

## Introduction

Head and neck cancer (HNC) refers to malignant tumours that occur in the area from the base of the skull to the supraclavicular and anterior cervical spine, including the neck, otorhinolaryngology and oral and maxillofacial parts (1), and it is currently the sixth most common malignant tumour and the seventh most common cause of tumour-related death worldwide (2). Radiotherapy serves as the sole treatment modality for early-stage head and neck tumors and plays a crucial role in the management of advanced-stage cases, where it is often combined with surgery or concurrent chemotherapy (3). Dysphagia is a common adverse effect due to radiotherapy for HNC (4). It is a process in which the patient is unable to effectively transport food into the stomach due to impaired structure and/or function of the jaw, tongue and lips, soft palate and pharynx (5). Approximately 30% to 80% of HNC patients can develop varying degrees of dysphagia after conventional radiotherapy (6–8), and also have adverse consequences including malnutrition, reduced psychosocial functioning, aspiration and aspiration pneumonia, and poor social participation and quality of life, which cause great harm to patients (9–11). The mechanism of dysphagia during radiotherapy is different from that of central nervous system damage in stroke patients. In HNC, many factors affect the structure and function of the swallowing organs, such as mucosal damage, pain, dry mouth due to irradiation, or muscle and soft tissue fibrosis and cricopharyngeal muscle narrowing as treatment progresses, resulting in decreased swallowing function (12, 13). The current state of fragmented and inadequate research, both domestically and internationally, hinders rapid access to comprehensive, scientific rehabilitation guidance for healthcare professionals. While domestic expert consensus on dysphagia is primarily used to guide stroke patients, there is limited evidence regarding the practice of rehabilitation management of dysphagia due to radiotherapy in HNC patients. To address this gap, our study will adopt an evidence-based approach to systematically search, collate, and summarize the evidence on the mechanism of dysphagia during radiotherapy in HNC patients. The goal is to provide scientific and effective evidence for the rehabilitation of swallowing function during radiotherapy in HNC patients, which is crucial for improving their quality of life.

## Materials and methods

### Retrieval strategy

The search was top-down according to the “6S” pyramid model and was based on a combination of free words and subject terms. English search terms “ ‘head and neck’ OR nasopharynx OR oropharynx OR larynx OR mouth OR hypopharynx” AND “tumor OR cancer OR neoplasm OR malignancy OR carcinoma” AND “dysphagia OR ‘deglutition disorder’ OR ‘swallowing disorder’ “ AND “care OR rehabilitation OR exercise OR physical therapy OR physical activity OR nursing”; Computer searches BMJ Best Practice, Up To Date, National Institute for Health and Clinical Excellence (NICE),Guidelines International Network(GIN), National Guideline Clearinghouse (NGC), Registered Nurses’ Association of Ontario (RNAO), Scottish Intercollegiate Guidelines Network (SIGN), Guidelines International Network(GIN), New Zealand Guidelines Group (NZGG), National Health and Medical Research Council (NHMRC), American Cancer Society (ACS), WHO Guidelines, China Guideline Clearinghouse (CGC), National Comprehensive Cancer Network (NCCN), Oncology Nursing Society (ONS), Medsci etc. guide network, and PubMed, Web of Science, CINAHL, Embase, Joanna Briggs Institute (JBI), Cochrane library, China Biology Medicine disc (CBM), China National Knowledge Infrastructure (CNKI), Wanfang Database etc. comprehensive database. The literature was searched for publications from January 2013 to March 2023. In addition, the references of the selected literature were searched for articles that contained expert consensus, evidence summaries, and best practice articles on swallowing rehabilitation for HNC patients during radiotherapy.

### Literature inclusion and exclusion criteria

Inclusion criteria were determined according to the evidence-based questions constructed by PIPOST: (i) the population to which the evidence was applied was HNC patients undergoing radiotherapy; (ii) the study content was rehabilitation measures for dysphagia; (iii) the implementers of the evidence were nurses, doctors, rehabilitation workers, nutritionists and other health promoters; (iv) the outcome indicators were swallowing function, nutritional status and swallowing-related quality of life; (v) the places where the evidence was applied were wards, rehabilitation centres and the community; (vi) the types of studies were guidelines, expert consensus, practice recommendations, clinical decision-making, evidence summaries, and systematic evaluations; (vii) the literature was published in Chinese or English. Exclusion criteria: (i) guidelines interpreted or directly translated; (ii) literature type was review; (iii) literature with incomplete information or full text not available; (iv) literature that has been updated; (v) duplicate publications.

### Literature screening and data extraction

Two researchers with systematic evidence-based training and experience in head and neck oncology independently screened the literature for inclusion and exclusion criteria by reviewing the titles, abstracts, and full texts. They extracted basic information and data from the selected studies and cross-checked the results. Any discrepancies were resolved through discussion with a third researcher to reach a consensus.

### Literature quality evaluation

Two researchers, who are Master’s students trained in a systematic evidence-based program at Fudan University, independently evaluated and graded the quality of evidence for inclusion. If any disagreements arise, a third investigator, who is a PhD candidate with extensive clinical and research experience, was sought to participate in decision-making and proofread the translation of the English evidence. 1) Guidelines: Guidelines were evaluated using the Appraisal of Guidelines for Research and Evaluation II (AGREE II) (14), which includes six domains: scope objectives, personnel involved, and development rigor, with 23 entries each representing a score of 1-7 from “strongly disagree” to “strongly agree”. Scores were standardized to the highest possible percentage of scores in the domain. 2) Expert consensus: the evaluation criteria developed by the JBI Center for Evidence-Based Health Care in its 2016 edition was used (15), which included six entries to label sources of opinion, reference to other literature, and state conclusions (16). 3) Clinical decision: evaluation was performed using an evaluation tool (critical appraisal for summaries of evidence, CASE) (17). 4) Practice recommendations, evidence summary: the original literature supporting their evidence was traced and evaluated for quality using the appropriate evaluation tool based on the type of literature. 5) Systematic reviews or Meta-analysis: the quality of the evaluation was assessed using the Assessment of Multiple Systematic Reviews (AMSTAR) tool (18), which includes 11 entries for evaluating evidence-based questions, search strategy, literature quality assessment, data extraction and synthesis, as well as publication bias (19).

### Evidence extraction, integration and evaluation

The content analysis method was used to extract evidence from the literature, which included general characteristics, research themes, and main contents of the literature. When evidence from different sources had complementary or consistent conclusions, a combined or general expression was used. However, if there were conflicting evidence from different sources, the principles of evidence-based priority, high-quality evidence priority, and latest published authoritative literature priority were followed. We graded the aggregated evidence using the Joanna Briggs Institute (JBI) Levels of Evidence and Grades of Recommendation system (2014 version) (20) from the Australian JBI Centre for Evidence-Based Healthcare. This system categorizes the evidence into five levels, from high to low, based on the study design of the included literature.

## Results

### General characteristics of included literatures

**Figure 1:**
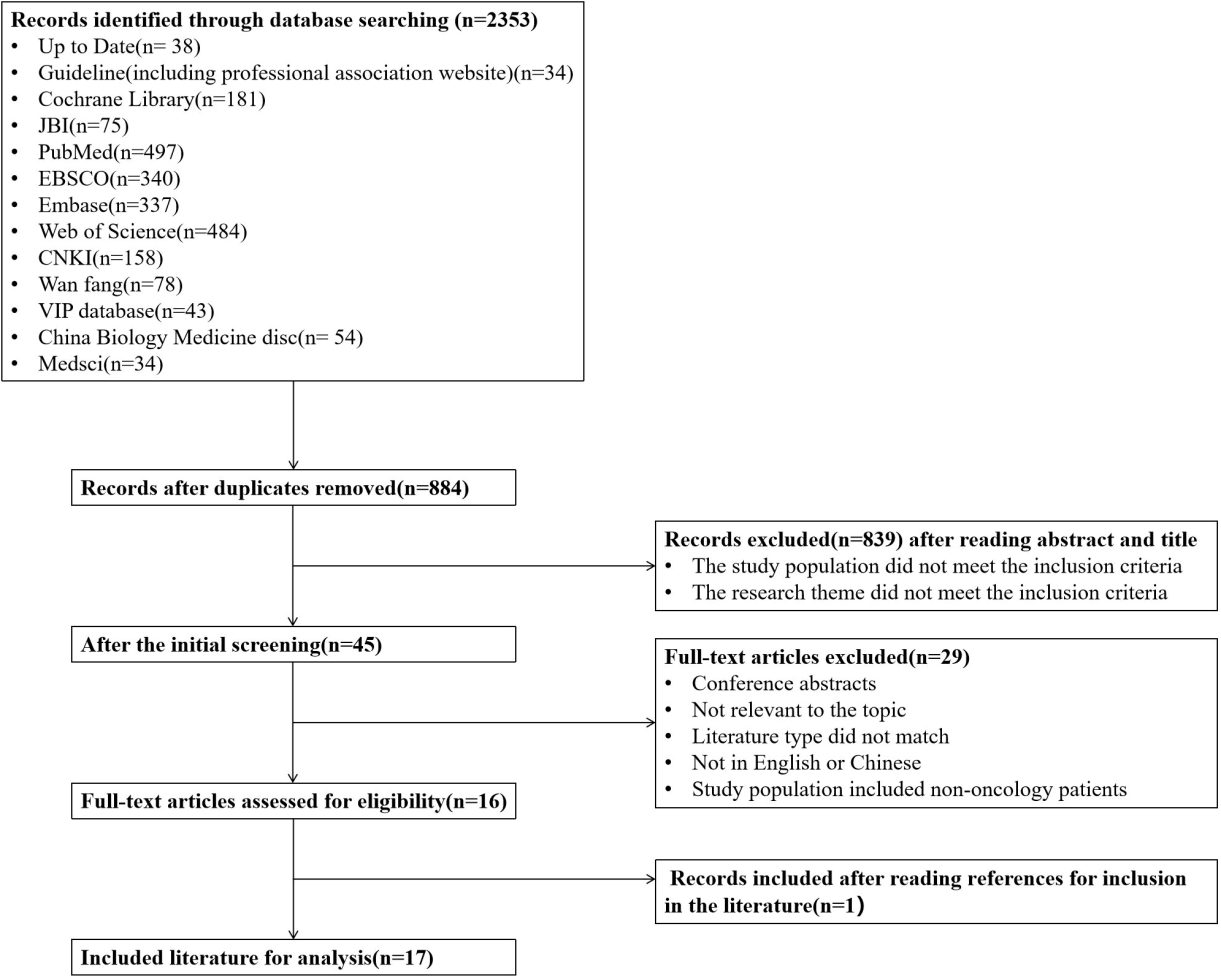
A flow chart of the literature screening process.

An initial search yielded a total of 2,353 articles, which were reduced to 1,469 after removing duplicates. Following a review of titles, abstracts, and full texts to eliminate non-compliant literature, 16 articles were included in the study, consisting of 3 guidelines, 5 expert consensus articles, 2 evidence summaries, 1 clinical decision, and 5 systematic reviews. One clinical practice recommendation for managing painful swallowing due to radiation oral mucositis was also extracted based on the topics covered and references cited in the included literature. In total, 17 articles were ultimately included. **Table 1** provides an overview of the basic characteristics of the literature included, while **Figure 1** shows a flowchart detailing the literature screening process.

**Table 1:** Characteristic of included literatures (n=17).

Included literatures	Source	Type of evidence	Topic	Year
Cohen (21)	ACS	Guideline	HNC Survival Care Guidelines	2016
Clarke (22)	PubMed	Guideline	Speech and Swallowing Rehabilitation Guidelines for HNC	2016
Cocks (23)	PubMed	Guideline	Guidelines for palliative and supportive care for HNC	2016
Goyal (24)	PubMed	Expert consensus	HNC Survival Consensus	2022
Schindler (12)	PubMed	Expert consensus	Consensus on dysphagia in patients with HNC treated with radiotherapy and systemic therapy	2015
Baijens (25)	PubMed	Expert consensus	European White Paper: Oropharyngeal Dysphagia in HNC	2020
Lin (26)	Medsci	Expert consensus	Nutritional interventions for HNC patients receiving concurrent radiotherapy	2018
Chong Zhao (27)	Medsci	Expert consensus	Expert consensus on nutritional and supportive care for patients undergoing radiotherapy for HNC	2021
Lewin (28)	UpToDate	Evidence Summary	Swallowing rehabilitation for HNC patients	2019
Jan (29)	UpToDate	Evidence Summary	Speech and swallowing rehabilitation for HNC patients	2021
Starmer (30)	PubMed	Clinical decision making	Clinical decision making in HNC patients with dysphagia	2019
Mirabile (31)	PubMed	Practice Recommendations	Pain management in radiotherapy HNC patients: clinical practice recommendations	2016
Brady (32)	PubMed	Systematic review	A systematic review of the impact of dysphagia prehabilitation on swallowing outcomes after radiotherapy for HNC	2017
Banda (33)	EBSCO	Systematic review	Swallowing exercises for patients with HNC: a systematic review and meta-analysis of RCTs	2021
Barbon (34)	PubMed	Systematic review	Efficacy of thickening fluids to eliminate aspiration in HNC: a systematic review	2015
Perry (35)	Cochrane Library	Systematic review	Therapeutic exercises affecting post-treatment swallowing in patients with advanced HNC	2014
Ren Xiaobo (36)	WanFang	Systematic review	Meta-analysis of the effect of swallowing training on swallowing function and quality of life in patients after radiotherapy for HNC	2022

HNC, head and neck; ACS, American Cancer Society; RCT, randomised controlled trial.

### Quality evaluation results of the included literature

#### Quality evaluation results of the guidelines

Three guidelines were included in this study. The guidelines were evaluated using AGREE II and the results are shown in **Table 2**, all with a recommendation level of A. The overall quality was high and inclusion was granted.

**Table 2:** Results of methodological quality evaluation included in the guidelines.

Guidelines	Percentage of field standardization %						≥60% field number (n)	≥30% field number (n)	Recommendation level
	Scope and purpose	Involved personnel	Preciseness of guidelines	Clarity of guidelines	Applicability of guidelines	Independence of guidelines			
Cohen (21)	90.0	81.3	86.0	77.3	68.4	78.6	6	6	A
Clarke (22)	90.0	88.8	85.5	78.2	85.0	71.1	6	6	A
Cocks (23)	86.7	78.8	83.3	73.5	82.2	75.0	6	6	A

#### Quality evaluation results of expert consensuses

Five expert consensus articles were included in this study. The quality evaluation criteria were evaluated using the JBI Center for Evidence-Based Health Care (2016) expert consensus evaluation criteria (15), and all entries were rated as “yes” and were included for overall high quality.

#### Quality evaluation results of clinical decision

One clinical decision was included in this study and was evaluated using the quality assessment tool CASE (17). The study by Starmer et al. (30) was evaluated as “partially yes” for “whether potential bias was avoided” and “yes” for the rest of the entries, which was of good overall quality and was included.

#### Quality evaluation results of practice recommendations and evidence summary

One practice recommendation and two evidence summaries were included in this study, which were evaluated for quality according to the type of primary literature using the appropriate evaluation tool. The overall quality was good and was included.

#### Quality evaluation results of systematic reviews

Five systematic reviews were included in this study and evaluated using the AMSTAR tool (18). In the study by Banda et al. (33), “Was a pre-design protocol provided?” was evaluated as “unclear”, while all other entries were evaluated as “yes”. In the study by Perry et al. (35), “Is a list of included and excluded studies provided?” was evaluated as “unclear” and the rest of the entries were evaluated as “yes”. In the study of Barbon et al. (34), “Do the inclusion criteria include the publication status of the literature, e.g. grey literature?”, “Do the conclusions drawn reasonably take into account the methodological quality of the included studies?”, “Was the potential for publication bias assessed” were evaluated as “unclear” and “Were the essential characteristics of the included studies described?” was evaluated as “no”, the rest of the evaluation results were “yes”. In the study by Brady et al. (32), all the entries were “yes” except “Is the method of combining results appropriate?” was evaluated as “no”. All the evaluation entries of the study by Ren et al. (36) were evaluated as “yes”, with high overall quality.

#### Summary and description of evidence

The evidence related to the 17 included literature will be extracted and summarised by this research team and discussed by two clinical nursing experts and two postgraduate students in clinical practice, resulting in a synthesis of evidence from six areas of swallowing function screening and assessment, physiotherapy, preventive swallowing function training, feeding management, pain control and oral care, resulting in 28 pieces of evidence, with the aim of providing healthcare professionals with better guidance for HNC patients Swallowing function exercises to prevent or alleviate dysphagia provide an evidence-based basis in **Table 3**.

**Table 3:** Summary of the evidence.

Category	Evidence content		Evidence level
**Screening and assessment of swallowing function**	**Assessors**	1. Screening and assessment emphasises a team approach, with nurses or head and neck oncologists recommended to screen patients with suspected swallowing problems, and speech-language pathologists to assess swallowing function in patients at risk of dysphagia (7, 21, 24, 29, 37)	2a
	**Timing of assessment**	2. Assess patients whenever they present with complaints such as dysphagia, postprandial cough, unexplained weight loss and/or pneumonia (12)	2a
		3. Patients are advised to undergo assessment of swallowing function and associated quality of life before, after and during the follow-up period of radiotherapy (12)	2a
	**Assessment tools**	4. VFSS and FEES are the gold standard for determining dysphagia (7, 12, 21, 25, 30) and are effective in predicting post-swallow residual volume, aspiration, aspiration pneumonia and pharyngeal stenosis due to radiotherapy (12, 21)	2b
		5. Different water swallowing tests are recommended as screening tools, e.g. 100ml WST, TOR-BSST, V-VST (25)	1a
		6. Use of targeted MDADI for HNC patients is recommended as an assessment tool for swallowing-related quality of life (12)	1b
	**Assessment content**	7. Speech and swallowing rehabilitation needs should be assessed prior to radiotherapy (22)	5b
		8. Clinical assessment includes oral motor examination (lip closure, range of motion), articulation, tongue control and tongue force, assessment of oropharyngeal swallowing (timing, efficiency, voice, tongue and larynx movements) and perception of voice quality (12, 22)	5b
		9. It is recommended that patients be monitored for aspiration, recurrent pneumonia and lung function during radiotherapy (12)	2a
**Physiotherapy**	10. sEMG with effortful swallow or Mendelsohn maneuver is recommended for patients with good compliance (24, 25)		1b
	11. Patients with cricopharyngeal muscle stenosis can use the catheter balloon dilation technique, which can be operated by doctors, nurses and speech therapists (25)		1a
**Prophylactic Swallowing Training**	12. Patients were encouraged to follow the speech pathologist’s instructions for preventive swallowing exercise, and nurses took on the role of rehabilitation therapists when there were not enough non-rehabilitation units and rehabilitation therapists (12, 22)		**5b**
	13. An active, individualised preventive swallowing function training programme should be formed for patients, with varying programme content, duration and frequency, and intervals (12)		**1a**
	14. Two types of exercise are recommended: one is airway protection methods such as the Mendelsohn maneuver, supraglottic swallow, super supraglottic swallow, and effortful swallow; the other is oral swallow training such as tongue strengthening exercises, Masako maneuver, and Shaker exercise (7, 12, 22, 36). Multiple swallowing training modalities are more effective when used in combination, with each movement held for 1-2s and then relaxed, and repeated 10 times a day (7)		**1b**
**Feeding management**	15. Assessment with NRS2002 at least once a week during radiotherapy, followed by PG-SGA for assessment and testing if there is a nutritional risk (27)		1a
	16. Prophylactic tube feeding for radiotherapy patients with HNC is controversial and is recommended taking into account the patient’s clinical condition, values and preferences (25, 28)		4a
	17. Patients with severe oral mucositis affecting swallowing function during radiotherapy are given enteral nutrition recommended as PEG or PEJ (27)		5b
	18. Regardless of the method of feeding (e.g. nasogastric tube, percutaneous endoscopic gastrostomy and parenteral nutrition), encourage patients to continue swallowing after tube placement and to disconnect from the feeding tube as soon as possible to ingest food via the mouth (12, 22)		5b
	19. Patients at risk of dysphagia should be screened and assessed for swallowing function before eating orally, and the assessment should include adjustments to food texture, texture and quantity, swallowing posture, eating tools and other means of compensating for oropharyngeal function (7, 22)		**5b**
	20. It is recommended that the viscosity of fluid foods be adjusted appropriately (30), that the order of feeding be paste-like foods first, with a gradual transition to soft rice equivalents after significant swallowing improvement (22, 25, 30), and that thickened liquids be consumed to reduce the risk of aspiration (22, 25, 30, 34)		**1a**
	21. It is recommended that an appropriate bite size and effective swallowing position be chosen after the V-VST or VFSS examination, with eating bites starting with a small amount (7, 30)		**1a**
**Pain management**	22. A variety of approaches are recommended to support patients with appropriate and personalised pain management (12, 30)		**5b**
	23. Regular use of pain assessment scales to assess swallowing-related pain (31)		**5b**
	24. Short-acting 2% lidocaine gargle (15-30 minutes) or long-acting morphine gargle (4-6 hours), or transdermal fentanyl or subcutaneous opioids are recommended to improve painful swallowing during radiotherapy (23, 31) 25. Prophylactic use of analgesics half an hour before eating improves swallowing (31)		**1b**
**Oral care**	26. Basic oral care can also reduce the frequency and extent of painful swallowing due to oral mucositis (31)		**1b**
	27. Removal of secretions from the patient’s mouth in a timely manner to avoid oral residues (25)		**5b**
	28. It is recommended to use a pH balanced mouthwash for gargling (30)		**1a**

VFSS, Video fluoroscopic swallowing; FEES, Fiberoptic endoscopic evaluation of swallowing; 100 ml WST, 100 ml Water Swallow Test; TOR-BSST, Toronto Bedside Swallowing Screening Test; V-VST, Volume-Viscosity Swallowing Test; MDADI, M.D. Anderson Dysphagia Inventory; sEMG, Surface electromyographic biofeedback; NRS 2002, nutrition risk screening 2002; PG-SGA, patient-generated subjective global assessment; PEG, Percutaneous Endoscopic Gastrostomy; PEJ, Percutaneous Endoscopic Jejunostomy.

## Discussion

This study provides evidence-based evidence on dysphagia to facilitate the rehabilitation of dysphagia in HNC patients undergoing radiotherapy by maximising the role of care. This study summarises the evidence related to swallowing rehabilitation for HNC patients undergoing radiotherapy in six areas: screening and assessment of dysphagia, physiotherapy, preventive swallowing function training, feeding management, pain control, and oral care, which may provide some guidance for patients’ clinical practice.

### Dysphagia screening and assessment facilitates early identification and management of swallowing function

Guidelines and consensus developed in different countries have successively provided recommendations on who should be screened and assessed for dysphagia, the purpose, timing and tools to facilitate early identification and management of dysphagia. The main aim of screening is to identify those at risk of dysphagia and to determine whether further investigations are needed (38). Therefore, it is generally recommended that nurses complete the screening of patients suspected of having swallowing problems, and other professionals may also be involved. It is also recommended that patients at risk of dysphagia should be referred to a speech and language therapist for a detailed swallowing assessment as soon as possible (7, 21, 24, 29, 37). Currently, several guidelines and consensus recommendations recommend the video fluoroscopic swallowing screening (VFSS) and fiberoptic endoscopic evaluation of swallowing (FEES) as the gold standard for the diagnosis of dysphagia (7, 12, 21, 25, 30). Although they are effective in predicting the onset of dysphagia in patients, they are costly, time-consuming and difficult to implement clinically (12, 38). Therefore, it should be clear what the purpose of the examination is with the intended message and it should not be misused. Researchers both domestically and internationally are continually seeking simpler and more convenient scales and assessment methods for screening and evaluating dysphagia. However, compared to the objective results obtained from patients who undergo FEES and VFSS, the sensitivity, specificity, and predictive values of subjective scales and other screening methods remain insufficient (12). Among the available screening tools, the M.D. Anderson Dysphagia Inventory (MDADI) is a relatively practical subjective dysphagia screening tool and is specifically designed for assessing swallowing-related quality of life in HNC patients, which is currently its primary application. However, the optimal method for screening dysphagia in HNC patients is yet to be determined.

### Physiotherapy methods for dysphagia are an effective way to improve dysphagia

Physiotherapy is commonly used to improve the physiological function of swallowing muscles in HNC patients who are undergoing radiotherapy, with the goal of achieving safe and effective swallowing. The most evidence-based physiotherapy treatments recommended by guidelines or consensus include surface electromyographic biofeedback (sEMG) training combined with catheter balloon dilation techniques (24, 25). sEMG training helps to enhance the strength and coordination of swallowing muscles under the guidance of a speech-language pathologist, and there is strong evidence that combined swallowing training is more effective (24, 25, 39). The catheter balloon dilation technique is a safe and reliable method to improve stenosis of the cricopharyngeal muscle caused by irradiation during radiotherapy, and can be performed by head and neck physicians, nurses, and speech-language pathologists (25, 39). While hand-held inductive electrical stimulation can also be used as an adjunct to swallowing therapy in radiotherapy patients, it is not widely available (39). Therefore, selection of appropriate physiotherapy treatment for dysphagia in HNC patients undergoing radiotherapy should be guided by professionals and based on the appropriate mechanism for this specific population.

### Prophylactic swallowing training facilitates the rehabilitation of swallowing function

Prophylactic swallowing training is a method to enhance the strength and coordination of muscle groups and improve the physiological function of swallowing. It enhances the strength of swallowing-related muscles and prevents the occurrence of dysphagia or reduces the severity of dysphagia by strengthening the control of jaw, lip and tongue movements, and soft palate and vocal cord closure movements (40, 41). Currently, rehabilitation functional training referred to in guideline and consensus relies heavily on specialist speech-language pathology (12, 22), with nurses often playing their role in non-rehabilitation units, less developed areas and where there are insufficient rehabilitation practitioners (39). Although prophylactic swallowing training is recommended in a number of guidelines and consensus studies, the content and efficacy of prophylactic swallowing training programmes for patients have not been standardised in current meta-analyses and systematic evaluations. Therefore, more studies with large samples of high-quality randomized controlled trials are needed (33, 35).

### Feeding management helps improve swallowing function and swallowing-related quality of life for patients

Nutrition is a primary concern for patients with dysphagia, and maintaining oral feeding has been shown to improve swallowing function and quality of life (42, 43). Some research suggests that nutritional support or prophylactic gastrostomy may benefit HNC patients undergoing treatment, whether or not chemotherapy is involved (12), use of these interventions should be based on the patient’s clinical status, values, and preferences (28). If a patient experiences severe oral mucositis that interferes with eating, enteral nutrition is often recommended to improve feeding (27). It is important to encourage patients to continue swallowing both before and after feeding tube placement, regardless of the method used (such as nasogastric tube, percutaneous endoscopic gastrostomy and parenteral nutrition) (12, 22).

Effective feeding management for patients with dysphagia requires consideration not only of the amount of nutrition, but also the method of food delivery, the nature of the food, and meal preparation (22). The national expert consensus recommends establishing a nutritional management team that includes a professional dietitian to screen patients for malnutrition risk during radiotherapy and whenever dysphagia is indicated. Additionally, compensatory oropharyngeal function, such as adjusting food properties, swallowing posture, and eating tools, is considered an important aspect of swallowing rehabilitation for patients who are able to swallow (7, 22). However, selection of appropriate food properties and swallowing posture for patients with dysphagia should be based on clinical and instrumental assessment (7, 30). Therefore, it is important to select evidence-based interventions tailored to the individual swallowing profile and preferences of HNC patients undergoing radiotherapy, to ensure safe and effective food intake and improve overall nutrition.

### Pain control helps maintain the patient’s normal swallowing process

For HNC patients during radiotherapy, mucosal and surrounding tissue pain in the irradiated area has a major impact on the patient’s swallowing process and compliance with swallowing function training (30). Some guidelines and consensus suggest that patients with dysphagia should be supported with appropriate and individualised pain management to reduce mucosal pain and maintain a normal swallowing process (12, 23), while disuse atrophy and fibrosis can be reduced and long-term swallowing function optimised (31). However, current guidelines and consensus do not give adequate recommendations for dysphagia due to mucosal pain in patients; therefore, this study was supplemented with evidence on the management of swallowing pain in HNC patients treated with radiotherapy by searching relevant reference searches of the included literature (31). Evidence 22 to 25 supplemented the evidence on the management of painful swallowing mainly in terms of pain assessment, timing of medication administration and basic oral care.

### Oral care helps to improve the safety and effectiveness of patients’ swallowing and improves their swallowing efficiency

Many HNC patients often experience sensory loss during radiotherapy due to mucosal and submucosal damage, resulting in inadequate cleaning of oral secretions and food debris. Consensus and clinical decision states that enhanced oral care can reduce the risk of aspiration of oral secretions and aspiration pneumonia (30, 39), while reducing the frequency and extent of painful swallowing and improving patients’ swallowing efficiency (31). Evidence recommends the use of pH-balanced mouthwashes for gargling in HNC patients during radiotherapy (30). Current recommendations for oral care for HNC patients with dysphagia during radiotherapy are inadequate and lack diversity, and it is recommended that more high-quality studies be conducted in the future to provide more reliable and targeted oral care options for patients.

## Conclusion

This study summarises the evidence on the rehabilitation management of HNC patients during radiotherapy and provides a reference for improving swallowing function, nutritional status and swallowing-related quality of life. The etiology and mechanisms of dysphagia during radiotherapy for HNC patients are complex, and there are geographical and cultural differences in the evidence across countries and regions in terms of ethnicity, values and healthcare delivery systems.
